# Edible exosome-like nanoparticles from portulaca oleracea L mitigate DSS-induced colitis *via* facilitating double-positive CD4^+^CD8^+^T cells expansion

**DOI:** 10.1186/s12951-023-02065-0

**Published:** 2023-08-31

**Authors:** Min-zheng Zhu, Hao-ming Xu, Yu-jie Liang, Jing Xu, Ning-ning Yue, Yuan Zhang, Cheng-mei Tian, Jun Yao, Li-sheng Wang, Yu-qiang Nie, De-feng Li

**Affiliations:** 1 Department of Gastroenterology and Hepatology, the Second Affiliated Hospital, School of Medicine, South China University of Technology, Guangzhou, 510006 Guangdong China; 2 School of Rehabilitation Medicine, Jining Medical University, Jining, 272029 Shandong China; 3 Department of Gastroenterology, Shenzhen People’s Hospital, the Second Clinical Medical College, Jinan University, Shenzhen, 518020 Guangdong China; 4 Department of Medical Administration, Huizhou Institute of Occupational Diseases Control and Prevention, Huizhou, 516000 Guangdong China; 5 Department of Emergency, Shenzhen People’s Hospital, the Second Clinical Medical College, the First Affiliated Hospital, Jinan University, Southern University of Science and Technology, Shenzhen, 518020 Guangdong China; 6 Department of Gastroenterology, Shenzhen People’s Hospital, the Second Clinical Medical College, the First Affiliated Hospital, Jinan University, Southern University of Science and Technology, Shenzhen, 518020 Guangdong China; 7 Shenzhen Clinical Research Centre for Geriatrics, Shenzhen People’s Hospital, Shenzhen, 518020 Guangdong China

**Keywords:** Portulaca oleracea L-derived exosome-like nanoparticles, *Lactobacillus reuteri*, Double-positive CD4^+^CD8^+^T, Ulcerative colitis

## Abstract

**Abstract:**

Plant-derived exosome-like nanoparticles (PDENs) have been paid great attention in the treatment of ulcerative colitis (UC). As a proof of concept, we isolated and identified Portulaca oleracea L-derived exosome-like nanoparticles (PELNs) from edible Portulaca oleracea L, which exhibited desirable nano-size (~ 160 nm) and a negative zeta potential value (-31.4 mV). Oral administration of PELNs effectively suppressed the expressions of pro-inflammatory cytokines (TNF-α, IL-6, IL-12, and IL-1β) and myeloperoxidase (MPO), increased levels of the anti-inflammatory cytokine (IL-10), and alleviated acute colitis in dextran sulfate sodium (DSS)-induced C57 mice and IL-10^−/−^ mice. Notably, PELNs exhibited excellent stability and safety within the gastrointestinal tract and displayed specific targeting to inflamed sites in the colons of mice. Mechanistically, oral administration of PELNs played a crucial role in maintaining the diversity and balance of gut microbiota. Furthermore, PELNs treatment enhanced *Lactobacillus reuteri* growth and elevated indole derivative levels, which might activate the aryl-hydrocarbon receptor (AhR) in conventional CD4^+^ T cells. This activation downregulated Zbtb7b expression, leading to the reprogramming of conventional CD4^+^ T cells into double-positive CD4^+^CD8^+^T cells (DP CD4^+^CD8^+^ T cells). In conclusion, our findings highlighted the potential of orally administered PELNs as a novel, natural, and colon-targeted agent, offering a promising therapeutic approach for managing UC.

**Graphic abstract:**

Schematic illustration of therapeutic effects of oral Portulaca oleracea L -derived natural exosome-like nanoparticles (PELNs) on UC. PELNs treatment enhanced *Lactobacillus reuteri* growth and elevated indole derivative levels, which activate the aryl-hydrocarbon receptor (AhR) in conventional CD4^+^ T cells leading to downregulate the expression of Zbtb7b, reprogram of conventional CD4^+^ T cells into double-positive CD4^+^CD8^+^T cells (DP CD4^+^CD8^+^ T cells), and decrease the levels of pro-inflammatory cytokines.

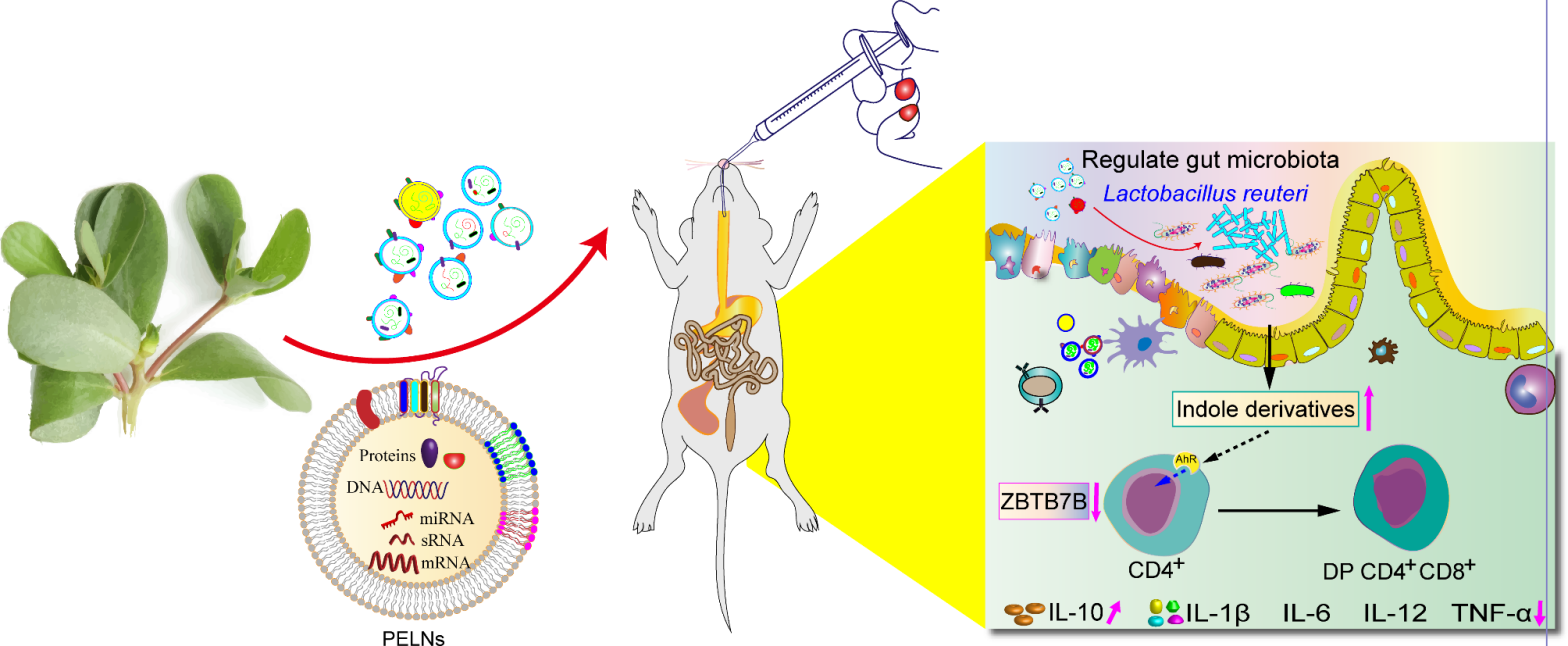

**Supplementary Information:**

The online version contains supplementary material available at 10.1186/s12951-023-02065-0.

## Introduction

Ulcerative colitis (UC) is an idiopathic inflammatory bowel disease (IBD) characterized by chronic damage to the colonic epithelial mucosa resulting in a relapsing and remitting course [1]. Clinical manifestations of UC include abdominal pain, diarrhea, blood in the stool, and weight loss [2]. Furthermore, UC has become a global disease with a steady upward trend worldwide, imposing a huge economic burden on healthcare systems [3]. In addition, patients with long-standing UC tend to develop colorectal cancer (CRC) [4]. Despite ongoing research, the precise etiology and pathogenesis of UC remain elusive. However, current evidence suggests that genetic, immunological, and environmental factors contribute to the development of UC [5–8]. The UC armamentarium mainly depends on non-targeted therapies, such as 5-aminosalicylates, glucocorticoids, and immunosuppressive agents, and targeted biologic therapies, such as anti-TNF antibodies [Infliximab (IFX), Adalimumab (ADL), Golimumab (GOLI), and Certolizumab pegol (CZP)], inhibitors of the p40 subunit of IL-12 and IL-23 (Ustekinumab), and JAK signaling pathway inhibitors (Tofacitinib) [7]. Unfortunately, intolerance, non-response, adverse events, and toxicity associated with these treatments hinder patients with UC from achieving remission [9]. Hence, there is an urgent need to develop novel medications with improved therapeutic efficacy and limited side effects.

Recently, there has been increasing interest in edible plant-derived exosome-like nanoparticles (PDENs), which are nanosized vesicles derived from edible plants, such as ginger, grape, carrot, and broccoli, and have been identified [10–13]. Accumulated studies have demonstrated that PDENs play a role in intercellular communication within plant cells and facilitate inter-kingdom communication due to their content of bioactive molecules, including lipids, proteins, and nucleic acids [14, 15]. PDENs offer several advantages of non-toxicity, low immunogenicity, and excellent biocompatibility [16–18]. These characteristics make PDENs highly promising for clinical applications in the treatment of various diseases, including UC. For example, grape-derived exosome-like nanoparticles have shown remarkable potential in promoting the proliferation of intestinal stem cells and facilitating mucosal epithelium regeneration, thereby inhibiting the progression of dextran sulfate sodium (DSS)-induced colitis in mice [11]. Additionally, oral administration of grape-derived exosome-like nanoparticles has demonstrated the ability to target intestinal epithelial cells (IECs) and macrophages, leading to increased survival and proliferation of IECs, decreased expression of pro-inflammatory cytokines, such as TNF-α, IL-6, and IL-1β, and elevated levels of anti-inflammatory cytokines, including IL-10 and IL-22, in experimental colitis induced by DSS [10]. Turmeric-derived exosome-like nanoparticles suppressed the expression of pro-inflammatory cytokines, such as TNF-α, IL-6, and IL-1β, promoted the levels of antioxidant genes, particularly HO-1 by inactivating NF-κB pathway, and alleviated colitis [19]. Broccoli-derived exosome-like nanoparticles inhibited the expression of pro-inflammatory cytokines, such as TNF-α, IL-17 A, and INF-γ, in experimental mouse colitis by inhibiting the activation of dendritic cells (DCs) activation and inducing tolerant DCs [20]. Orally administered gingle-derived exosome-like nanoparticles promote the survival and proliferation of intestinal epithelial cells (IECs), inhibit the expression of pro-inflammatory cytokines (TNF-α, IL-6, and IL-1β), and increase the levels of anti-inflammatory cytokines (IL-10 and IL-22) contributing to relieve colitis [10]. A randomized controlled trial is currently underway to investigate the effectiveness and safety of grape-derived exosome-like nanoparticles in patients with UC [21]. Importantly, PDENs hold great promise as a viable alternative for the treatment of UC.

A growing body of evidence indicates that PDENs play a pivotal role in modulating the composition and localization of intestinal microbiota, as well as maintaining intestinal microbiota homeostasis in UC treatment. For instance, tea-derived exosome-like nanoparticles treatment has been shown to significantly enhance species diversity and community abundance of intestinal microbiota compared to the DSS control group in mice model of colitis [22]. Furthermore, treatment with tea-derived exosome-like nanoparticles has been found to decrease the *Firmicutes/Bacteroidetes* ratio and the abundance of *Oscillibacter*, while increasing the populations of *Bifidobacterium*, *Lachnospiraceae*, and *Akkermansia*, which promotes intestinal microbiota homeostasis in mice with colitis [22]. In another study, orally administered ginger-derived exosome-like nanoparticles exhibit preferential uptake by *Lactobacillaceae* in intestinal microbiota, and specifically target specific genes of *Lactobacillus rhamnosus* (LGG), contributing to the alteration in the composition of gut microbiota and the increased levels of IL-22, thereby restoring intestinal barrier function and mitigating mice colitis [23]. Additionally, oral administration of mulberry bark-based exosome-like nanoparticles promote the abundance of *Firmicutes*, *Proteobacteria*, and *Verrucomicrobia*, while decreasing the community richness of *Actinobacteria*, *Bacteroidetes* and *Tenericutes*, which reshape the maintenance of intestinal microbiota in an experimental colitis model [24].

Portulaca oleracea L. (purslane, POL) is a widely used medicinal herb with a cosmopolitan distribution and extensively utilized in many countries [25]. Numerous studies have highlighted the diverse pharmacological effects of POL, including anti-inflammatory, antioxidant, immune-regulating, and antitumor activities [26]. Additionally, POL has been shown to effectively attenuate DSS-induced colitis by inhibiting oxidative stress responses, such as nitric oxide and superoxide dismutase, as well as reducing the levels of pro-inflammatory cytokines like TNF-α, IL-1β, and IL-6 [27–29]. In this study, we presented the first isolation and purification of natural exosome-like nanoparticles derived from POL using ultracentrifugation and sucrose gradient centrifugation. Subsequently, we demonstrated that oral administration of POL-derived exosome-like nanoparticles (PELNs) selectively targeted inflammatory sites in DSS-induced mice UC model. Furthermore, PELNs effectively alleviated colitis by suppressing the expression of pro-inflammatory cytokines and promoting the secretion of anti-inflammatory cytokines. Mechanistically, PELNs enhanced the growth of *Lactobacillus reuteri* and increased indole derivatives levels, which led to the downregulation of Zbtb7b expression and subsequently induced the differentiation of double-positive CD4^+^CD8^+^T cells (DP CD4^+^CD8^+^ T cells) in mice with colitis.

## Methods

### Isolation and purification of PELNs from POL

The POL samples were thoroughly washed with running water and then placed into phosphate-buffered saline (PBS). Subsequently, the POL was cut into small pieces and homogenized using a blender. To eliminate plant fibers and large particles, a low-speed centrifugation step (500-3,000 g for 10–15 min) was employed. This was followed by a medium-speed centrifugation step (3,000–10,000 g for 20–40 min) to remove larger debris and intact organelles. Finally, a high-speed centrifugation step (10,000-150,000 g for 1.5-2 h) was performed to obtain a pellet containing PELNs. The pellet was resuspended in sterile PBS and subjected to purification using a sucrose gradient (8%, 30%, 45%, and 60% sucrose in 20 mM Tris-Cl pH 7.2). The purification process involved centrifugation at 150,000 g for 2 h at 4 °C using an SW41 rotor (Beckman Coulter, Fullerton, CA, USA). Fractions with PELNs of specific density were combined, and the resulting mixture was concentrated via ultracentrifugation (100,000 g for 4 h, 4 °C). The band observed in the 45/60% sucrose layer was collected and subjected to three washes. The resulting extracellular vesicle (EV) pellet was then resuspended in PBS and stored at -80 °C until further use (Figure S1a).

### Physicochemical characterization

PELNs samples were subjected to analysis using NanoSight NS300 technology (Malvern Instruments, Malvern, United Kingdom) to determine the size and concentration distribution of the nanoparticles. The zeta potential of PELNs was determined using dynamic light scattering (DLS) with a system from Malvern Instruments (Malvern, UK). Cryo-electron micrographs of the PELNs samples were obtained using a transmission electron microscope Glacios (Thermo Fisher Scientific, USA), equipped with a Ceta camera. The microscope was operated at an accelerating voltage of 200 kV. Grid mapping and image acquisition were performed using EPU software (Thermo Fisher Scientific, USA) at a nominal magnification of ×8,500. High-magnification images were recorded at a nominal magnification of ×73,000 (0.2 nm pixel size) with a defocus value of -3.0 μm. To minimize radiation damage during image acquisition, the electron dose was controlled to be below 30 e-/Å-2.

### Lipidomic analysis

The lipid extraction was carried out using the Bligh and Dyer method. Briefly, 100 µg of PELNs was added to a 10-mL glass tube. A mixture of methanol and dichloromethane (2 mL methanol: 0.9 mL dichloromethane, v/w) was added to the tube, followed by rotation for 30 s. The sample was then incubated at room temperature for 30 min. Next, high-performance liquid chromatography (HPLC)-grade water and 0.9 mL dichloromethane were added to the tube. The sample was gently inverted 10 times and then centrifuged at 1,200 rpm for 10 min. The organic lower phase (dichloromethane) was collected, concentrated under nitrogen to dryness, and reconstituted in methanol: dichloromethane (1:1, v/w) containing 10 mM ammonium acetate.

The extracted lipid was analyzed using a Waters Acquity UPLC system coupled with a Thermo Fisher Q Exactive quadrupole Orbitrap mass spectrometer. Separation of lipids was performed using an ACQUITY UPLC BEH C18 column (2.1*100 mm, 1.7 μm particle size). Ultrahigh-performance liquid chromatography was carried out with a 16-minute gradient. Lipids were detected in positive and negative ion modes, and the mass spectrometer was operated in first-stage full scan (Full Scan, m/z range: 200-1,200) and data-dependent secondary mass spectrometry scanning (dd-MS2, TopN = 10) acquisition modes. The acquired data were then processed using Thermo Scientific LipidSearch software. The software performed qualitative analysis using the lipid database, and the peak areas were integrated to determine the relative content of lipids.

### In vitro stability of PELNs

A stomach-like solution was prepared by combining 18.5% (w/v) HCl (pH 2.0) and pepsin solution (80 mg/mL in 0.1 N HCl, pH 2.0, Sigma). Additionally, 4 mg/mL of pancreatin (Sigma) was added to the solution. This stomach-like solution (1 mL, 1 mg/mL) was mixed with PELNs in PBS and incubated at 37 °C for 4 h. To create a small intestine-like solution, the pH of the stomach-like solution was adjusted to approximately 6.5 using 1 N NaHCO_3_. This adjusted solution served as a small intestine-like environment. PELNs were then incubated in the small intestine-like solution for an additional 60 min. The stability of PELNs in these solutions was assessed by measuring the particle size and surface charge using the methods described earlier.

### Animals

C57BL/6 and IL-10^−/−^ mice, both aged 9 weeks and weighing 25–26 g, were obtained from a specific pathogen-free (SPF) facility (Biotechnology Co., Ltd., Beijing, China). The mice were housed in a pathogen-free environment with a 12-hour light/dark cycle. All animal experiments were conducted in accordance with the guidelines and regulations of the Animal Care Committee of Shenzhen People’s Hospital, Shenzhen, China, and received approval from the committee.

### In vivo therapeutic outcomes of PELNs against colitis in mice

C57BL/6 male mice were randomly divided into four groups: the healthy control group, the DSS group, the DSS + 50 mg/g PELNs-L group, and the DSS + 100 mg/g PELNs-H group. All mice were given 3% DSS in their drinking water continuously for 7 days to induce colitis. Mice in the PELNs treatment groups received oral gavage with the respective PELNs dosage once daily for 5 days, starting from day 2 of the DSS treatment. Throughout the experiment, the body weight, fecal characteristics, and physical activity of the mice were monitored on a daily basis. Disease severity scores were calculated according to a previously established scoring system. On day 7, the mice were euthanized using CO_2_ inhalation. Blood samples were collected from the orbits of the mice for enzyme-linked immunosorbent assay (ELISA). Colon tissues, feces, blood serum, and major organs (heart, liver, spleen, lung, and kidney) were harvested for further examinations.

IL-10^−/−^ mice were randomly assigned to three groups: the healthy control group, the DSS group, and the oral administration PELNs-H group, based on the findings obtained from previous experiments. The therapeutic effect of PELNs on DSS-induced colitis in IL-10^−/−^ mice was assessed using the same methodology described earlier.

### PELNs labeling and stability

For labeling PELNs, IRDye 800CW near-infrared fluorescent dyes (IRDye® 800CW NHS Ester) were utilized. In brief, a 0.21-mM solution of fluorescent IRDye 800CW was added to 1 mg of PELNs in 1 mL of PBS. The mixture was then incubated with a 0.2-M sodium bicarbonate buffer (pH 8.3) for 2 h at room temperature. Subsequently, the labeled PELNs were subjected to a 100-kDa ultracentrifuge filter to remove any free dye. The resulting IRDye® 800CW-labeled PELNs were suspended in PBS for further experiments.

To investigate the behavior of the IRDye® 800CW-labeled PELNs in different conditions, they were incubated in a stomach-like solution or a small intestine-like solution at 37 °C for 30 min. Following the incubation, the IRDye® 800CW-labeled PELNs were collected using exosome spin columns with a molecular weight cutoff of 4,000 (MW4000).

### Biodistribution of orally administrated PELNs

To investigate the in vivo biodistribution of PELNs, a mouse model of UC was orally administered with IRDye 800CW-labeled PELNs at a dosage of 100 mg/g. The aim was to track the distribution of PELNs in the gastrointestinal tract (GIT). At various time points after oral administration (3, 6, 12, and 24 h), the mice were euthanized using CO_2_ inhalation. Colon tissues were then harvested for fluorescence imaging using an IVIS spectrum imaging system (Hopkinton, USA). This imaging system allows for visualization and quantification of the fluorescence emitted by the IRDye 800CW-labeled PELNs in the colon tissues.

### 16 S rDNA sequencing

#### Extraction of fecal DNA and analysis of gut microbiota

After sacrificing the mice, the intestinal contents were carefully removed from the colon. These contents were immediately frozen with liquid nitrogen and transferred to a -80 °C refrigerator for subsequent DNA extraction and microbial analysis. The HiPure Stool DNA Kit from Magen (Guangzhou, China) was used to extract total fecal DNA from the frozen intestinal contents. The primers used for amplification were as follows: 341 F: CCTACGGGNGGCWGCAG and 806R: GGACTACHVGGGTATCTAAT. The amplified products were purified using the Phusion High-Fidelity PCR Master Mix from New England Biolabs (Beverly, USA). Sequencing libraries were generated using the TruSeq DNA PCR-Free Sample Preparation Kit from Illumina (San Diego, USA). The quality of the constructed libraries was assessed using the Agilent Bioanalyzer 2100 system and the Qubit@ 2.0 Fluorometer from Thermo Scientific (Carlsbad, USA). The Illumina HiSeq 2500 platform, operated by Tianjin Novogene Bioinformatics Technology Co., Ltd., was utilized for sequencing the libraries [30].

### 16 S rDNA bioinformatics analyses

#### Operational taxonomic units (OTUs) analysis

The effective tags of OTUs with a similarity threshold of 97% were clustered together using UPARSE software (version 9.2.64) [31]. In this process, each cluster was represented by the most abundant tag sequence within that cluster. The resulting OTUs, including exclusive OTUs (unique to each group) and shared OTUs (common across multiple groups), were analyzed and visualized using the R project (version 2.2.1). An updated plot was generated to display the number of OTUs, exclusive OTUs, and shared OTUs for each group.

### Diversity analysis

To assess the α-diversity of the microbial communities, various diversity indices, including the ACE index, Chao1 index, Simpson index, Shannon index, and PD-whole tree index, were calculated using QIIME (version 1.9.1) [32]. PD-whole tree index was calculated in picante (version 1.8.2) [33]. To compare the α-indices among the three groups, Tukey’s honestly significant difference (HSD) test was employed. Additionally, Welch’s t-test was applied to evaluate the differences between the two groups. For β-diversity analysis, the VennDiagram package (version 1.4.4) [34] in the R project was utilized to analyze the distribution of elements. Principal component analysis (PCA), principal coordinates analysis (PCoA), and non-metric multidimensional scaling (NMDS) of Bray-Curtis distances were performed using the Vegan package [35, 36] in the R project. These multivariate statistical techniques were used to analyze and visualize the dissimilarity of microbial communities among the samples.

### Community composition analysis

We utilized a naive Bayesian model implemented through RDP classifier [37] (version 2.2) with reference to the SILVA [38] databases for sequence classification according to organisms. To represent the community composition, a stacked bar plot was generated using the ggplot2 package (version 2.2.1) within the R project.

### Welch’s t test

To assess significant differences in mean abundance between the two groups, Welch’s t-test was employed. This statistical test was selected due to the following criteria being met: [1] each group had a minimum of three repeat samples, and [2] at least one sample exhibited a species tag count equivalent to or exceeding 0.1% of the total tags.

### RNA extraction and qRT-PCR

The RNA extraction and qRT-PCR procedures were conducted as previously described [39]. Colon samples were subjected to RNA extraction using the TRIzol Reagent (Thermo, USA). The concentration of RNA was determined using the NanoDrop 2000 (Thermo Scientific, Massachusetts, USA). Reverse transcription was performed using the PrimeScript RT Master Mix (Takara Biomedical Technology, Beijing, China). The primer sequences utilized are provided in Table S1.

### Enzymelinked immunosorbent assay (ELISA)

The concentrations of IL-6, IL-12, IL-1β, TNF-α, IL-10, and myeloperoxidase (MPO) in serum tissue were determined using commercial ELISA kits, following the manufacturer’s instructions (Elabscience Biotechnology Co., Ltd.).

### Flow cytometry (FCM)

FCM was employed to analyze the population of DP CD4^+^CD8^+^ T cells in the colon samples. The procedure was conducted as previously described [39].

### Immunofluorescence (IF)

IF was performed to detect the population of DP CD4^+^CD8^+^T cells in the colon samples as previously described [39].

### Western blotting (WB) analysis and immunohistochemistry (IHC) assay

WB analysis and IHC assay were performed as previously described [39]. An Antibodies specific to Zbtb7b were acquired from Proteintech (Cat.: 11341-1-AP, Wuhan, China), while antibodies targeting GAPDH were sourced from Affinity Biosciences (Cat.: AF7021, Jiangsu, China). Additionally, goat anti-rabbit IgG antibody was obtained from Southern Biotech (Cat.: 4050-05 A, Labama, USA). Antibody dilutions for Zbtb7b were 1:1,000 for WB analysis and 1:100 for IHC assay.

### Growth Curve Experiment of *Lactobacillus reuteri*

*Lactobacillus reuteri* (BNCC 192190) (BeNa Culture Collection, China) was incubated overnight in MRS medium at 37℃ within an anaerobic chamber. Subsequently, the PELNs (20 mg/µL) were added as a supplement. The control group was defined as the culture without PELNs. The growth curve was determined by measuring turbidity at 600 nm using the Multiskan-Go spectrophotometer (Thermo Fisher Scientific, Vantaa, Finland) at 37℃ every 2 h.

### LC-MS/MS

To prepare the resuspension solution, a vacuum freeze-dried fecal sample weighing 50 mg was homogenized using a homogenization procedure (65 Hz, 3 min). The homogenized sample was mixed with 900 µL of an extracting solution consisting of methanol and ultrapure water in a 1:9 ratio (v/v). Following that, vacuum concentration was performed at 45℃ for 3 h. The resulting solution was resuspended with 200 µL of the extracting solution, followed by centrifugation at 15,000 × g for 10–15 min. The obtained supernatant was then filtered using a 0.22-µm membrane to acquire the supernatant for LC-MS/MS analysis.

### Statistical analysis

A comparison of multiple experimental groups was carried out by one-way or two-way ANOVA. A t test was calculated to compare the means of the two groups. The p values < 0.05*, P < 0.01**, P < 0.001***, P < 0.0001**** were considered to be statistically significant.

## Results

### Characterization of PELNs

The PELNs were isolated from the POL, and found to accumulate abundantly at the 8/30% interface (band 1) and the 30/45% interface (band 2) of the sucrose gradient, as depicted in Figure S1a. The size distribution of PELNs exhibited characteristics similar to exosome-like nanoparticles, as confirmed by electron microscopic examination (Figure S1b). Furthermore, hydrodynamic particle size analysis revealed a range of 30 to 400 nm for PELNs, with an average size of 180 nm (Figure S1c). Zeta potential measurements demonstrated that PELNs exhibited a negative zeta potential of -31.4 mV (Figure S1d). The lipidomic analysis indicated that PELNs primarily consisted of digalactosyldiacylglycerol (DGDG, 24.47%), triglyceride (TG, 17.09%), and phosphatidylcholine (PC, 9.28%) (Figure S1e).

To assess the stability of PELNs in the GIT, they were subjected to incubation in different aqueous solutions simulating stomach-like and small intestine-like environments. Changes in their zeta potential and size were analyzed. The zeta potential of PELNs exhibited a decrease in negative charge in the stomach-like solution, whereas a weak positive change was observed in the small intestine-like solution (Figure S1f). The results also revealed an increase in heterogeneity in size when incubated in both stomach-like and small intestine-like solutions, as compared to water incubation for 2 h (Figure S1g). Remarkably, these findings suggested that PELNs could maintain their integrity and resist digestion during transit through the GIT.

Additionally, IRDye® 800CW-labeled PELNs were subjected to incubation in aqueous solutions simulating a stomach-like and a small intestine-like environment to evaluate their stability. The results demonstrated that the fluorescent signals emitted by IRDye® 800CW-labeled PELNs were not weakened after incubation in both the stomach-like and small intestine-like solutions (Figure S1h and S1i). This observation indicated that the acidic and alkaline environments in the stomach and intestine did not disrupt the linkage between the exosomes and the dye, ensuring the stability of the labeling.

### Oral administration of PELNs protects mice against DSS-induced colitis

**Fig. 1:**
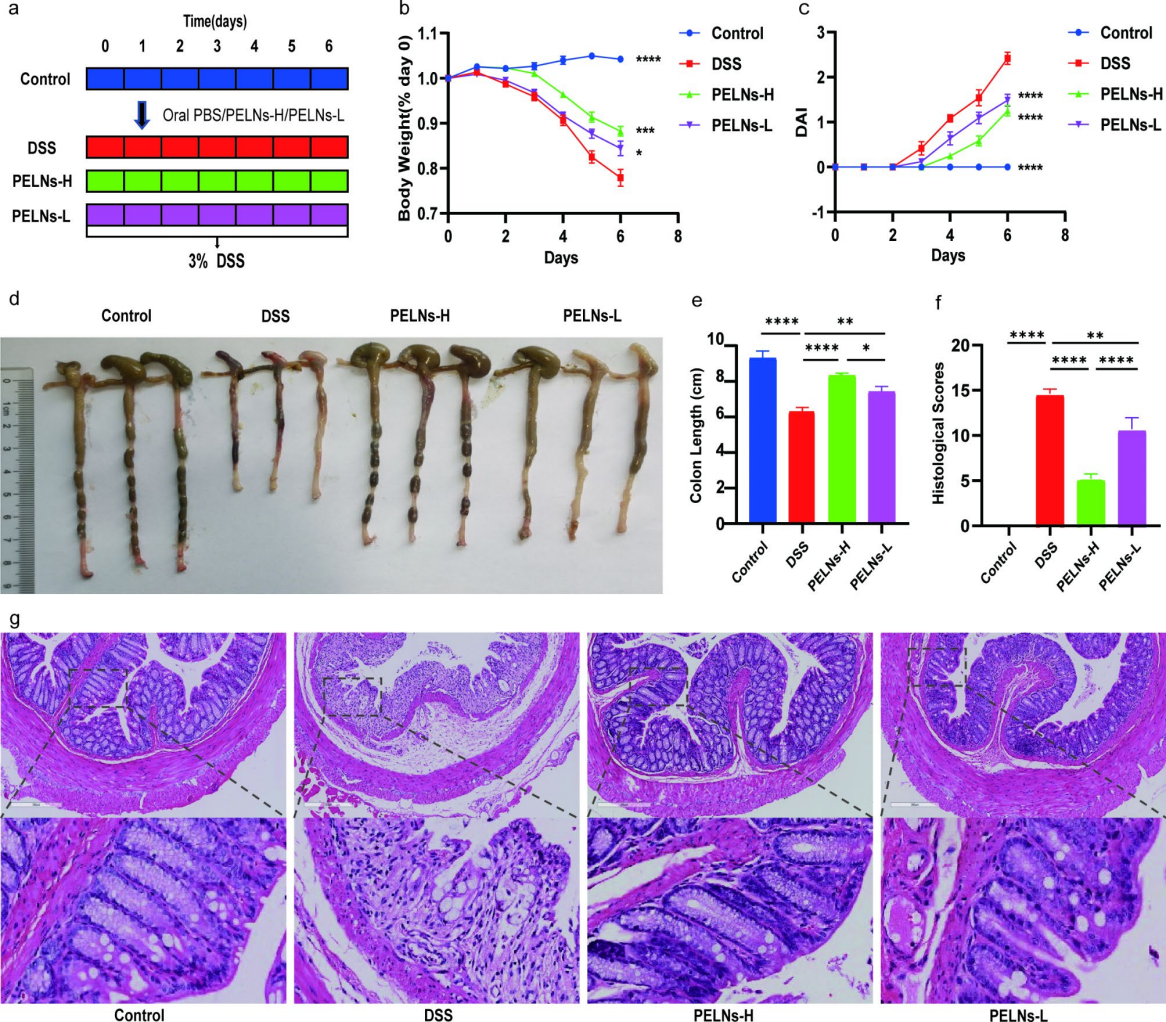
Oral administration of PELNs protects mice against DSS-induced colitis. **a**, Protocol for DSS-induced colitis and PELNs administration; **b**, Changes of body weight over time, normalized to the percentage of the day-zero body weight; **c**, Disease activity index (DAI); **d, e**, Colon length; **f**, Histological scores; **g**, H&E-stained colon sections. *P < 0.05, **P < 0.01, ***P < 0.001 ****P < 0.0001

To investigate the anti-inflammatory effects of PELNs, mice were divided into four groups: a healthy control group, a DSS control group, a PELNs-L group, and a PELNs-H group, as described in Fig. 1a. Throughout the experiment, the body weight of mice in the healthy control group was gradually increased, while a significant decrease was observed in the DSS control group (Fig. 1b). However, treatment with PELNs showed potential in preventing weight loss compared to the DSS control group (Fig. 1b). Notably, PELNs-H appeared to be more effective in preventing weight loss compared to PELNs-L (Fig. 1b). Similarly, PELNs played a crucial role in reducing the disease activity index (DAI) (Fig. 1c). The PELNs groups exhibited significantly lower DAI compared to the DSS control group (Fig. 1c), and the DAI in the PELNs groups was comparable to that in the healthy control group (Fig. 1c). Additionally, PELNs significantly prevented colon shortening (Fig. 1d and e), as the colon length of the PELNs groups was longer compared to the DSS control group (Fig. 1d and e). Moreover, the colon length in the PELNs-H group was slightly longer than that in the PELNs-L group, and the fecal condition and mucosa of the colon in the PELNs-H group appeared better than that in the PELNs-L group, although still shorter than in the healthy control group (Fig. 1d and e). Histological analysis, including hematoxylin-eosin staining (H&E) and histological scores, further confirmed that PELNs reduced the accumulation of immune cells and damage to the colonic epithelial barrier (Fig. 1f and g). Interestingly, the histological scores were significantly lower in the PELNs-H group compared to the PELNs-L group (Fig. 1g), indicating that PELNs-H had a more potent anti-inflammatory therapeutic effect than PELNs-L in the treatment of DSS-induced colitis in mice.

**Fig. 2:**
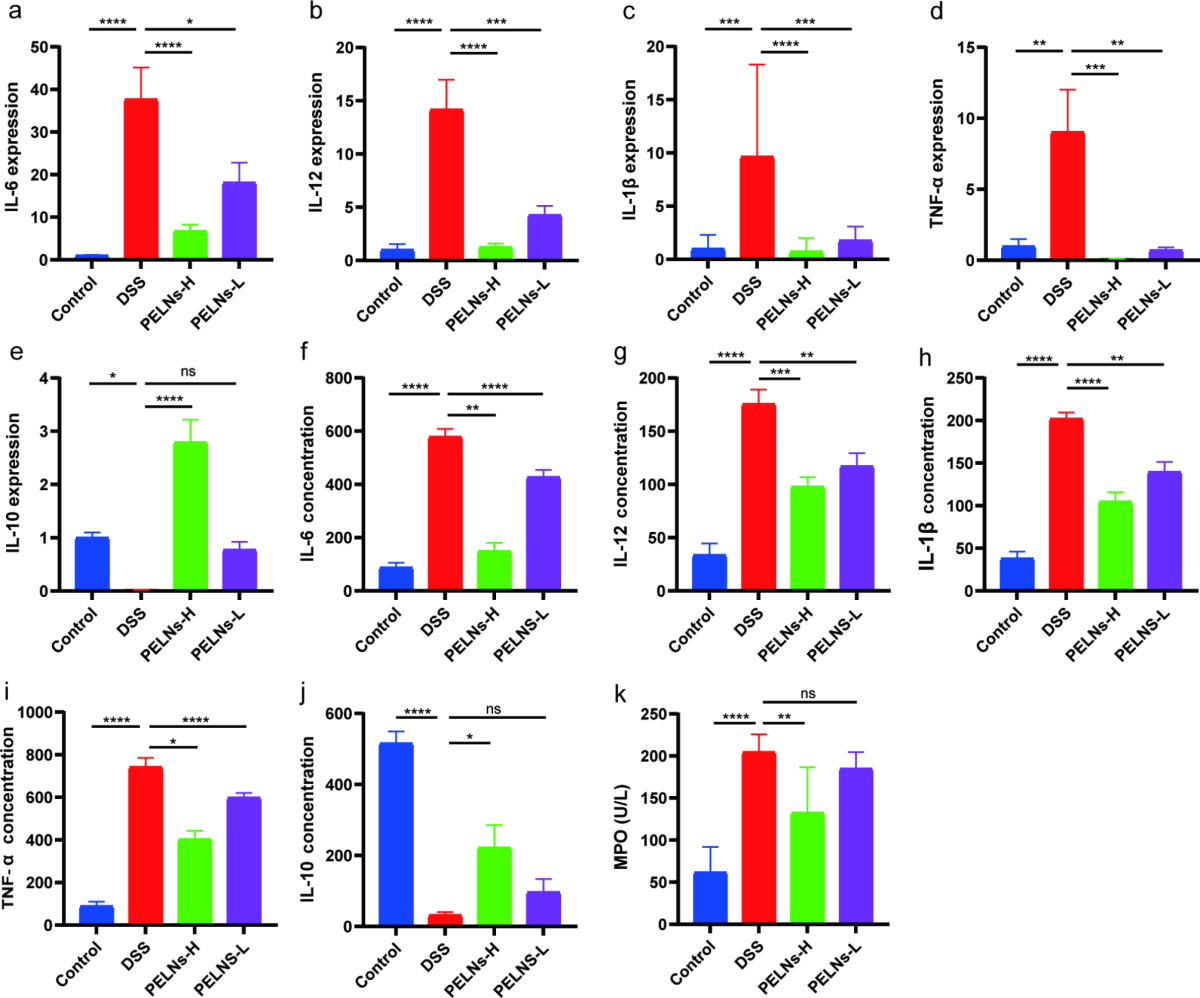
Pro-inflammatory cytokines and anti-inflammatory cytokine expression profiles. **a, b, c, d, e**, qRT-PCR detecting the levels of IL-6, IL-12, IL-1β, TNF-α and IL-10 in colonic samples; **f, g, h, i, j, k**, ELISA testing the expression profiles of IL-6, IL-12, IL-1β, TNF-α, IL-10 and MPO in blood serum. *P < 0.05, **P < 0.01, ***P < 0.001 ****P < 0.0001

It is known that pro-inflammatory cytokines play a crucial role in intestinal inflammation. In colon samples, the levels of pro-inflammatory cytokines (IL-6, IL-12, IL-1β, and TNF-α), were significantly elevated in the DSS control group compared to the healthy control group, as determined by qRT-PCR (Fig. 2a, b, c and d). However, treatment with PELNs remarkably inhibited the expression of these pro-inflammatory cytokines (Fig. 2a, b, c and d). Conversely, IL-10, an anti-inflammatory cytokine, exhibited a considerable decrease in the DSS control group compared to the healthy control group, while its levels were significantly increased in the PELNs groups (Fig. 2e).

Furthermore, an ELISA conducted on blood samples demonstrated a significant increase in the secretion levels of pro-inflammatory cytokines (IL-6, IL-12, IL-1β, and TNF-α), as well as myeloperoxidase (MPO), a marker associated with neutrophils in the DSS control group compared to the healthy control group (Fig. 2f g, 2 h, 2i, and 2k). However, treatment with PELNs significantly reduced the secretion levels of these pro-inflammatory cytokines and MPO (Fig. 2f g, 2 h, 2i, and 2k). Similarly, the secretion level of IL-10 was measured and revealed that PELNs promoted the secretion of IL-10 (Fig. 2j). Notably, PELNs-H exhibited better performance in decreasing pro-inflammatory cytokines and increasing anti-inflammatory cytokines compared to PELNs-L in the treatment of mouse colitis.

### Oral administration of PELNs protects IL-10^−/−^ mice acute colitis

**Fig. 3:**
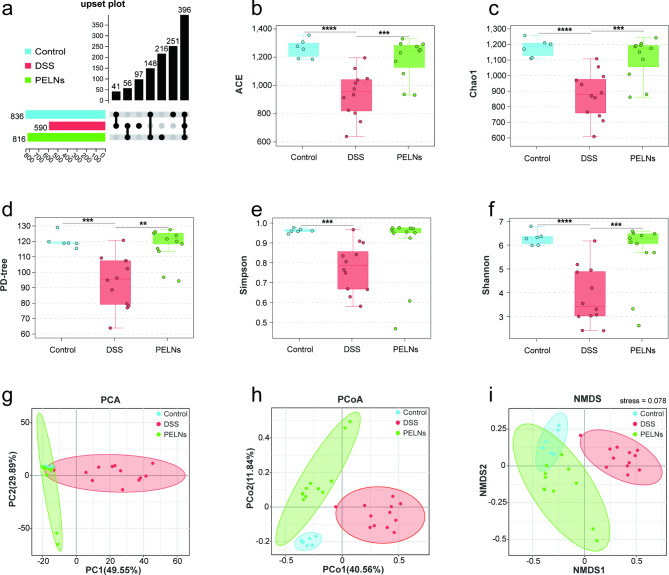
Oral administration of PELNs protects IL-10^−/−^ mice spontaneous colitis. **a**, Changes of body weight; **b**, Disease activity index (DAI); **c**, Colon length; **d**, Histological scores; **e**, H&E-stained colon sections

To further assess the anti-inflammatory effects of PELNs in an acute colitis model, IL-10^−/−^ mice were fed and divided into three groups: healthy control group, DSS control group, and PELNs-H group. Surprisingly, IL-10^−/−^ mice treated with PELNs-H failed to develop acute colitis. PELNs-H treatment effectively prevented body weight loss, DAI, colon shortening, reduced histological scores and immune cell infiltration compared to IL-10^−/−^ mice not treated with PELNs-H (Fig. 3a, b, c and d, and 3e).

Moreover, qRT-PCR and ELISA results revealed that oral administration of PELNs-H effectively suppressed the expression of pro-inflammatory cytokines (IL-6, IL-12, IL-1β, and TNF-α), as well as MPO concentration in both colon tissues and blood samples of IL-10^−/−^ mice with acute colitis (Figure S2). These findings highlighted the potent anti-inflammatory properties of PELNs-H in mitigating colitis symptoms in the IL-10^−/−^ mouse model.

### In vivo distribution of PELNs

**Fig. 4:**
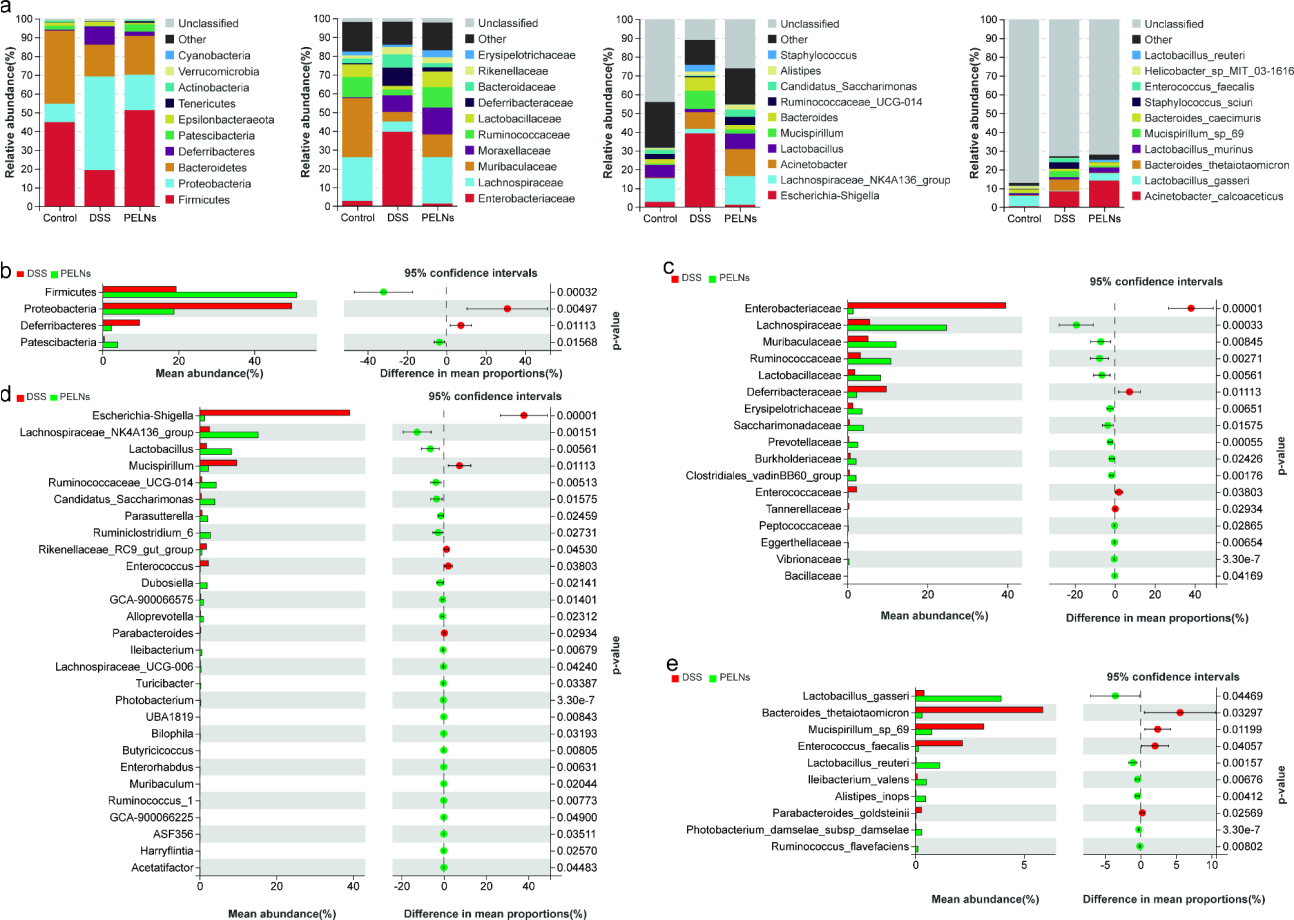
In vivo distribution of PELNs. Fluorescence images of the gastrointestinal tract revealing in vivo bio-distribution of orally administered IRDye 800CW-labeled PELNs at different time points (3, 6, 12, and 24 h) in acute colitis mice (**a**) and health mice (**b**)

The accumulation of oral nanotherapeutics in the desired colon is crucial for exerting therapeutic effectiveness against UC. In order to determine the biodistribution of PELNs after oral administration, mice with UC were gavaged with IRDye 800CW-labeled PELNs (100 mg/g) for 3, 6, 12, and 24 h. Near-infrared imaging revealed that IRDye 800CW-labeled PELNs were visibly present in the colon at 3 h, and their presence was gradually increased at 6 h in colitis mice. However, the fluorescent signals detected in the colon were steadily decreased by 24 h (Fig. 4a). Notably, low fluorescence signals were detected in the colon when PELNs were administered to healthy mice (Fig. 4b). Subsequently, the accumulation of fluorescent signals in vital organs, such as the heart, liver, spleen, lung, and kidney, was examined. It was found that very few fluorescent signals were detected in these organs in mice with colitis treated with PELNs and health control at 3, 6, 12, and 24 h (Fig. 4a and b). This finding indicated that PELNs selectively targeted the inflamed colon in the treatment of colitis in mice.

### PELNs distinctly altered the diversity of the intestinal microflora

**Fig. 5:**
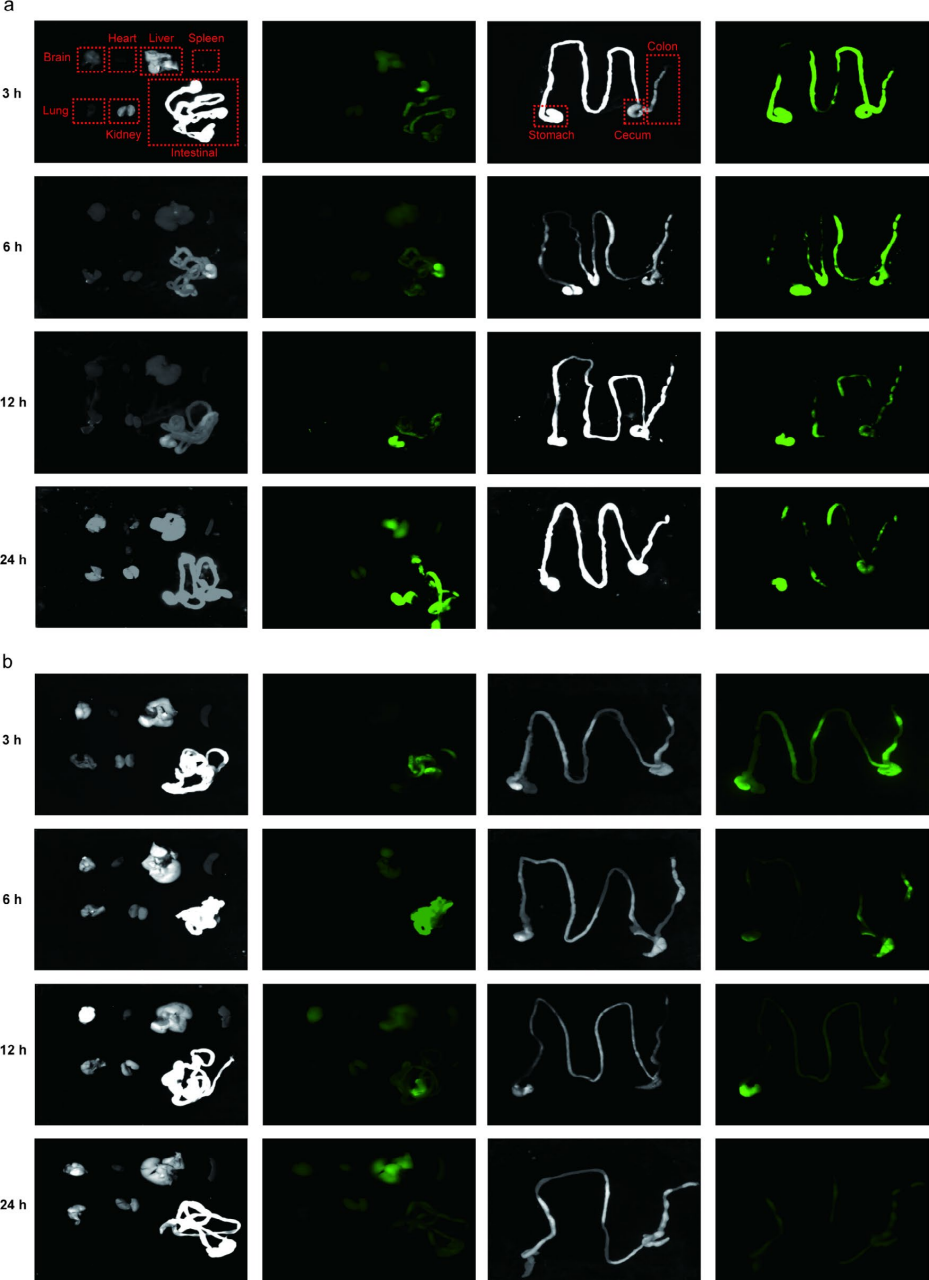
PELNs alters diversity of gut microbiota in mice models of colitis. **a**, Upset Polt of OTUs; **b**, ACE index; **c**, Chao 1 index; **d**, PD-tree index; **e**, Simpson index; **f**, Shannon index; **g**, PCA (Principal Component Analysis); **h**, PCoA (principal co-ordinate analysis). Sum of PCoA1 and PCoA2 more than 50%, indicating significant difference; **i**, NMDS (Non-metric Multi-Dimensional Scaling). Stress value less than 0.1, indicating significant difference. **P < 0.01, ***P < 0.001, ****P < 0.0001

The profiles of intestinal microbiota were analyzed using 16 S rDNA sequencing. The abundance of gut microbiota was assessed by calculating the number of OTUs, which was significantly increased after PELNs treatment compared to colitis mice, as depicted in Fig. 5a. To further evaluate the effect of PELNs on α-diversity, five diversity indices were examined: ACE (Fig. 5b), Chao1 (Fig. 5c), PD-tree (Fig. 5d), Simpson (Fig. 5e), and Shannon (Fig. 5f). DSS-induced colitis led to a significant decrease in α-diversity as indicated by all five indices (ACE, P = 0.00002; Chao1, P = 0.000016; PD-tree, P = 0.000171; Simpson, P = 0.000257; Shannon, P = 0.000015). However, treatment with PELNs significantly increased α-diversity, as reflected by the upregulation of the ACE (P = 0.000775), Chao1 (P = 0.000707), PD-tree (P = 0.001010), Simpson (P = 0.107145), and Shannon (P = 0.003565) indices. β-diversity was assessed to examine the differences between the gut microbiota of different groups using PCA (Fig. 5g), PCoA (Fig. 5h), and NMDS (Fig. 5i). Consistent results were observed across all three methods. The NMDS, PCA (PCA1 + PCA2 = 79.44 > 50%), and PCoA (PCoA1 + PCoA2 = 52.40 > 50%) analyses showed that PELNs significantly altered the microbial structure of colitis mice, bringing it closer to the healthy control group. Additionally, the stress value of the NMDS analysis was 0.078, indicating a good fit to the data. Overall, these findings suggested that PELNs treatment modulated the composition and structure of the intestinal microbiota in colitis mice, leading to increased α-diversity and restoration of the microbial structure towards a healthier state.

### PELNs improve DSS induced changes in dominant microbiota and mediated significant changes in microbial structure

**Fig. 6:**
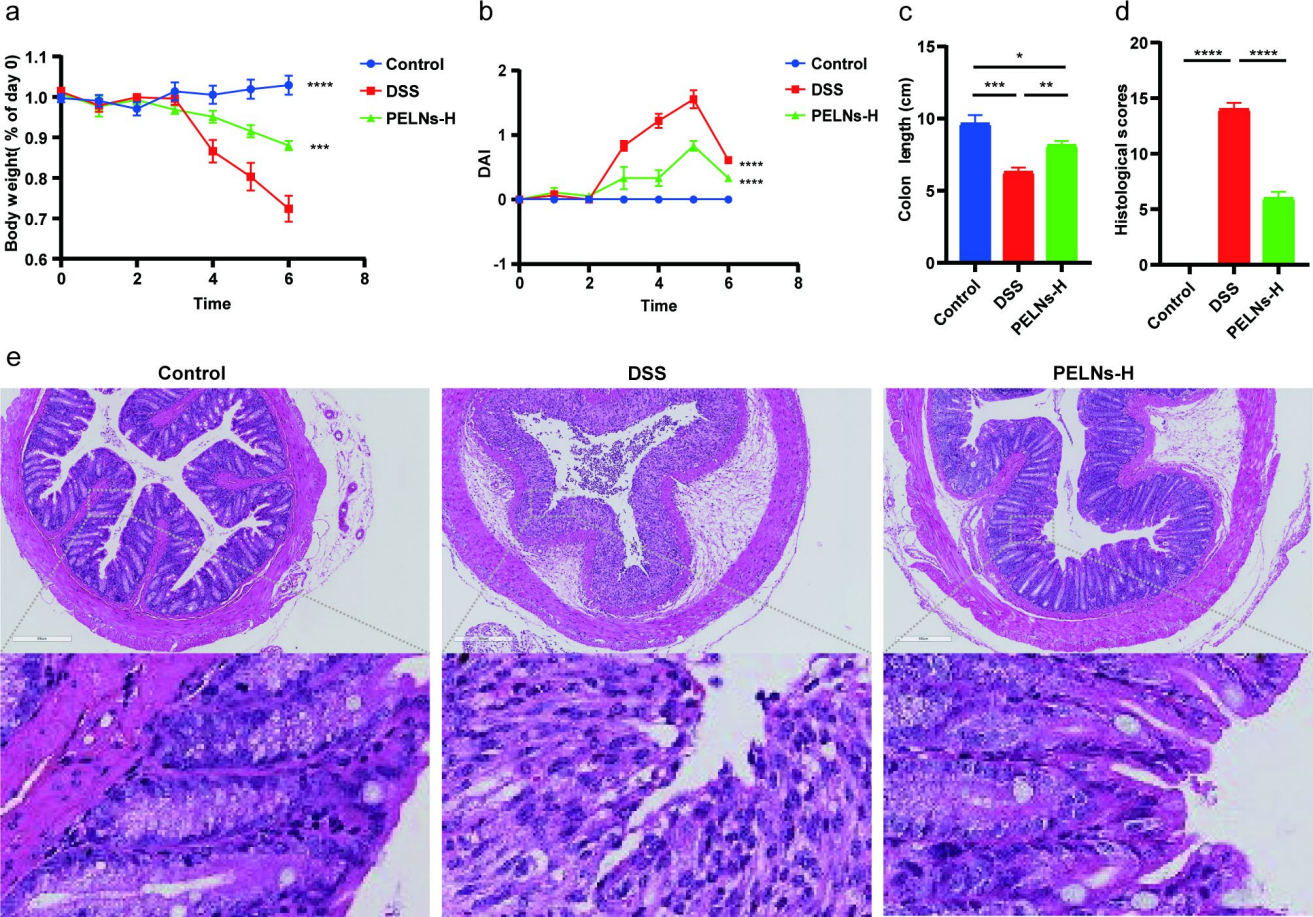
PELNs alters gut microbiota structures across different levels in mice with colitis. **a**, Stacked bar plot depicts the structure of gut microbiota in each group of mice. Left to right: phylum, family, genus, and species; **b-e**, Welch’s t test analysis of PELNs mediated differential microbial changes at the phylum, family, genus, and species level

In Fig. 6a, the relative abundance of certain bacterial taxa was significantly different between the mice treated with PELNs and the colitis group. *Firmicutes* (51.26% vs. 19.36%, P = 0.000141), *Patescibacteria* (3.92% vs. 0.41%, P = 0.006606), *Tenericutes* (0.92% vs. 0.10%, P = 0.089697), *Actinobacteria* (0.46% vs. 0.13%, P = 0.071755), *Lachnospiraceae* (24.75% vs. 5.53%, P = 0.000056), *Ruminococcaceae* (10.81% vs. 3.18%, P = 0.001079), *Muribaculaceae* (12.08% vs. 5.09%, P = 0.008093), *Lactobacillaceae* (8.23% vs. 1.81%, P = 0.002318), *Moraxellaceae* (14.34% vs. 8.82%, P = 0.502718), *Erysipelotrichaceae* (3.64% vs. 1.32%, P = 0.004203), *Lachnospiraceae_NK4A136_group* (15.23% vs. 2.53%, P = 0.000263), *Lactobacillus* (8.22% vs. 1.81%, P = 0.002315), *Acinetobacter* (14.34% vs. 8.82%, P = 0.502718), *Ruminococcaceae_UCG-014* (4.30% vs. 0.51%, P = 0.001301), *Candidatus_Saccharimonas* (3.92% vs. 0.41%, P = 0.006644), *Alistipes* (2.61% vs. 2.02%, P = 0.514264), *Acinetobacter_calcoaceticus* (14.11% vs. 8.43%, P = 0.484667), *Lactobacillus_gasseri* (3.95% vs. 0.40%, P = 0.027309), *Lactobacillus_murinus* (2.80% vs. 1.30%, P = 0.177553), *Lactobacillus_reuteri* (1.12% vs. 0.03%, P = 0.000201), and *Bacteroides_caecimuris* (1.82% vs. 1.20%, P = 0.531045) were notably elevated in the PELNs-treated mice. Conversely, the abundances of *Proteobacteria* (18.79% vs. 49.81%, P = 0.004250), *Deferribacteres* (2.28% vs. 9.67%, P = 0.010716), *Epsilonbacteraeota* (0.76% vs. 2.15%, P = 0.188453), *Verrucomicrobia* (0.10% vs. 0.42%, P = 0.221802), *Cyanobacteria* (0.05% vs. 0.09%, P = 0.253980), *Enterobacteriaceae* (1.37% vs. 39.56%, P < 0.0001), *Deferribacteraceae* (2.28% vs. 9.67%, P = 0.010716), *Bacteroidaceae* (2.31% vs. 7.14%, P = 0.070016), *Rikenellaceae* (3.19% vs. 3.83%, P = 0.631456), *Escherichia-Shigella* (1.27% vs. 39.13%, P < 0.0001), *Mucispirillum* (2.28% vs. 9.67%, P0.010716=), *Bacteroides* (2.31% vs. 7.14%, P = 0.070016), *Staphylococcus* (0.17% vs. 3.67%, P = 0.164005), *Bacteroides_thetaiotaomicron* (0.31% vs. 5.87%, P = 0.029962), *Staphylococcus_sciuri* (0.12% vs. 3.61%, P = 0.162046), *Mucispirillum_sp_69* (0.75% vs. 3.15%, P = 0.011562), and *Enterococcus_faecalis* (0.13% vs. 2.17%, P = 0.037956) were decreased.

Welch’s t-test was employed to examine the microbial changes induced by PELNs. The analysis revealed a higher abundance of *Firmicutes* and *Patescibacteria* (Fig. 6b), while *Proteobacteria* and *Deferribacteres* exhibited decreased abundance. Notably, the relative proportion of several families (Fig. 6c), including *Lachnospiraceae*, *Ruminococcaceae*, *Muribaculaceae, Lactobacillaceae*, *Saccharimonadaceae*, *Erysipelotrichaceae*, *Prevotellaceae*, *Clostridiales_vadinBB60_group*, *Burkholderiaceae*, *Vibrionaceae*, *Eggerthellaceae*, *Peptococcaceae*, and *Bacillaceae*, showed a remarkable increase. On the other hand, the abundances of *Enterobacteriaceae*, *Deferribacteraceae*, *Enterococcaceae*, and *Tannerellaceae* were decreased compared to the colitis group. Furthermore, at the genus level (Fig. 6d), the PELNs group exhibited higher relative abundances of *Lachnospiraceae_NK4A136_group*, *Lactobacillus*, *Ruminococcaceae_UCG-014*, *Candidatus_Saccharimonas*, *Ruminiclostridium_6*, *Dubosiella*, *Parasutterella*, *Alloprevotella*, *GCA-900,066,575*, *Ileibacterium*, *Photobacterium*, *Turicibacter*, *Lachnospiraceae_UCG-006*, *Ruminococcus_1*, *Bilophila*, *Enterorhabdus*, *Butyricicoccus*, *ASF356*, *UBA1819*, *Harryflintia*, *GCA-900,066,225*, *Muribaculum*, and *Acetatifactor*, while *Escherichia-Shigella*, *Mucispirillum*, *Enterococcus*, *Rikenellaceae_RC9_gut_group*, and *Parabacteroides* exhibited lower abundances. At the species level (Fig. 6e), the relative proportions of *Lactobacillus_gasseri*, *Lactobacillus_reuteri*, *Alistipes_inops*, *Ileibacterium_valens*, *Photobacterium_damselae_subsp_damselae*, and *Ruminococcus_flavefaciens* were drastically elevated by PELNs, whereas *Bacteroides_thetaiotaomicron*, *Mucispirillum_sp_69*, *Enterococcus_faecalis*, and *Parabacteroides_goldsteinii* showed decreased proportions. (The Welch’s t-test results comparing the control group to the DSS group can be found in the supplementary materials, Figure S3).

### PELNs-*Lactobacillus reuters*-indole-derivatives axis induces the differentiation of DP CD4^+^CD8^+^T cells

**Fig. 7:**
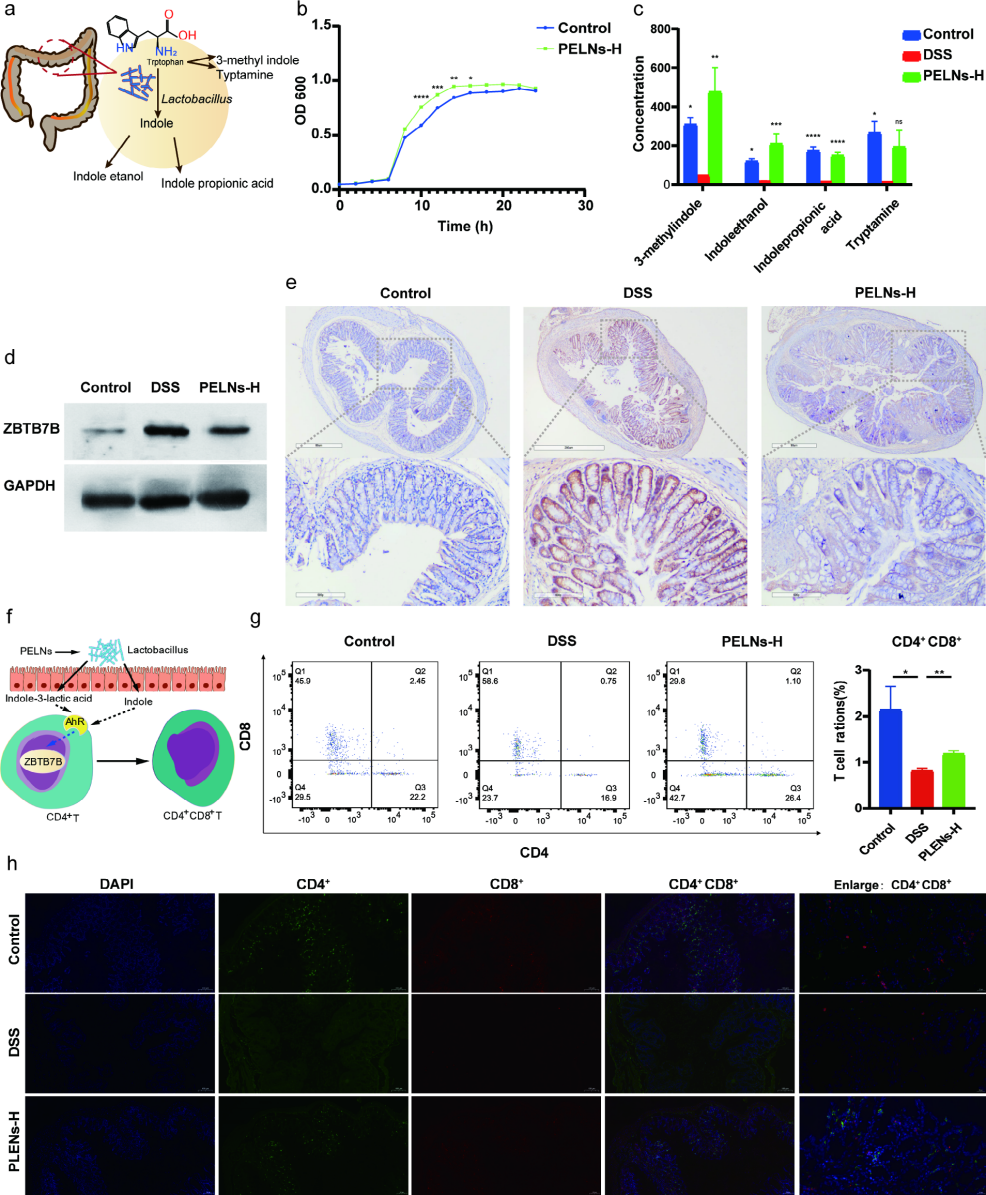
PELNs inducing the differentiation of DP CD4^+^CD8^+^T cells. **a**, Schematic representation of mechanisms for *Lactobacillus reuteri* metabolizing tryptophan (L-Trp) into indole derivatives; **b**, PELNs promote the growth of *Lactobacillus reuteri*; **c**, PELNs treatment significantly increased the levels of indole derivatives; **d, e**, PELNs treatment significantly decreased the levels of Zbtb7b protein using WB and IHC assay; **f**, Schematic representation for the derivatives of *Lactobacillus reuteri* activating the aryl hydrocarbon receptor (AhR) in conventional CD4^+^ T cells, and leading to the downregulation of Zbtb7b expression and the reprogramming of conventional CD4^+^ T cells into DP CD4^+^CD8^+^ T cells; **g**, Flow cytometry detecting the population of DP CD4^+^CD8^+^ T cells in colonic samples; **h**, Represent immunofluorescence showing the population of DP CD4^+^CD8^+^ T cells in colonic samples. Data are representative FCM images or expressed as the mean ± SEM of each group. *P < 0.05, **P < 0.01

It has been discovered that *Lactobacillus reuteri* had the ability to metabolize tryptophan (L-Trp) into indole derivatives. These derivatives could activate the aryl hydrocarbon receptor (AhR) in conventional CD4^+^ T cells, leading to the downregulation of Zbtb7b expression and the reprogramming of conventional CD4^+^ T cells into DP CD4^+^CD8^+^ T cells (Fig. 7a and f) [37]. To assess the growth behavior of *Lactobacillus reuteri*, OD_600_ measurements were performed in vitro with and without PELNs. The results demonstrated that while the growth of isolates was comparable between the PELNs group and the control group during the stationary and delay periods, the growth of isolates was noticeably faster in the logarithmic phase in the PELNs group compared to the control group (Fig. 7b). Additionally, PELNs treatment significantly increased the levels of indole derivatives, including 3-methylindole, indoleethanol, indolepropionic acid and tryptamine, in fecal samples as compared to the DSS group (Fig. 7c and Figure S4).

DP CD4^+^CD8^+^ T cells are localized in the intestinal epithelial layer and serve multiple functions, including the suppression of intestinal inflammation induced by type 1 helper T (Th1) cells, maintenance of gut mucosal homeostasis, and inhibition of pro-inflammatory cytokine release during pathogenic infections [37]. It has also been observed that the down-regulation of Zbtb7b in intraepithelial T lymphocytes (IELs) represses CD4^+^ T cell differentiation while promoting the differentiation of CD8^+^ T cells, leading to the reprogramming of CD4^+^ T cells into DP CD4^+^CD8^+^ T cells [37]. In our previous studies, we have demonstrated a significant increase in Zbtb7b expression and a notable decrease in the population of DP CD4^+^CD8^+^ T cells in both UC patients and UC mouse colon samples [36]. Therefore, we aimed to investigate the impact of oral administration of PELNs on the differentiation of DP CD4^+^CD8^+^ T cells in mice with colitis. Strikingly, we observed a significant reduction in the expression of Zbtb7b at the protein level in the PELNs group, as demonstrated by WB analysis and IHC assay (Fig. 7d and e). Additionally, the population of DP CD4^+^CD8^+^ T cells was significantly higher in the PELNs group compared to the DSS control group, as determined by FCM (1.1% vs. 0.75%, P < 0.01, Fig. 7g). However, there was a significant difference in the population of DP CD4^+^CD8^+^ T cells between the PELNs group and the healthy group (1.1% vs. 2.45%, P < 0.05, Fig. 7g). Similarly, IF analysis revealed a marked increase in the population of DP CD4^+^CD8^+^ T cells in the PELNs group compared to the DSS control group (Fig. 7h). Furthermore, the population of DP CD4^+^CD8^+^ T cells in the PELNs group was comparable to that of the healthy group (Fig. 7h).

### Biosafety of orally administered PELNs

**Fig. 8:**
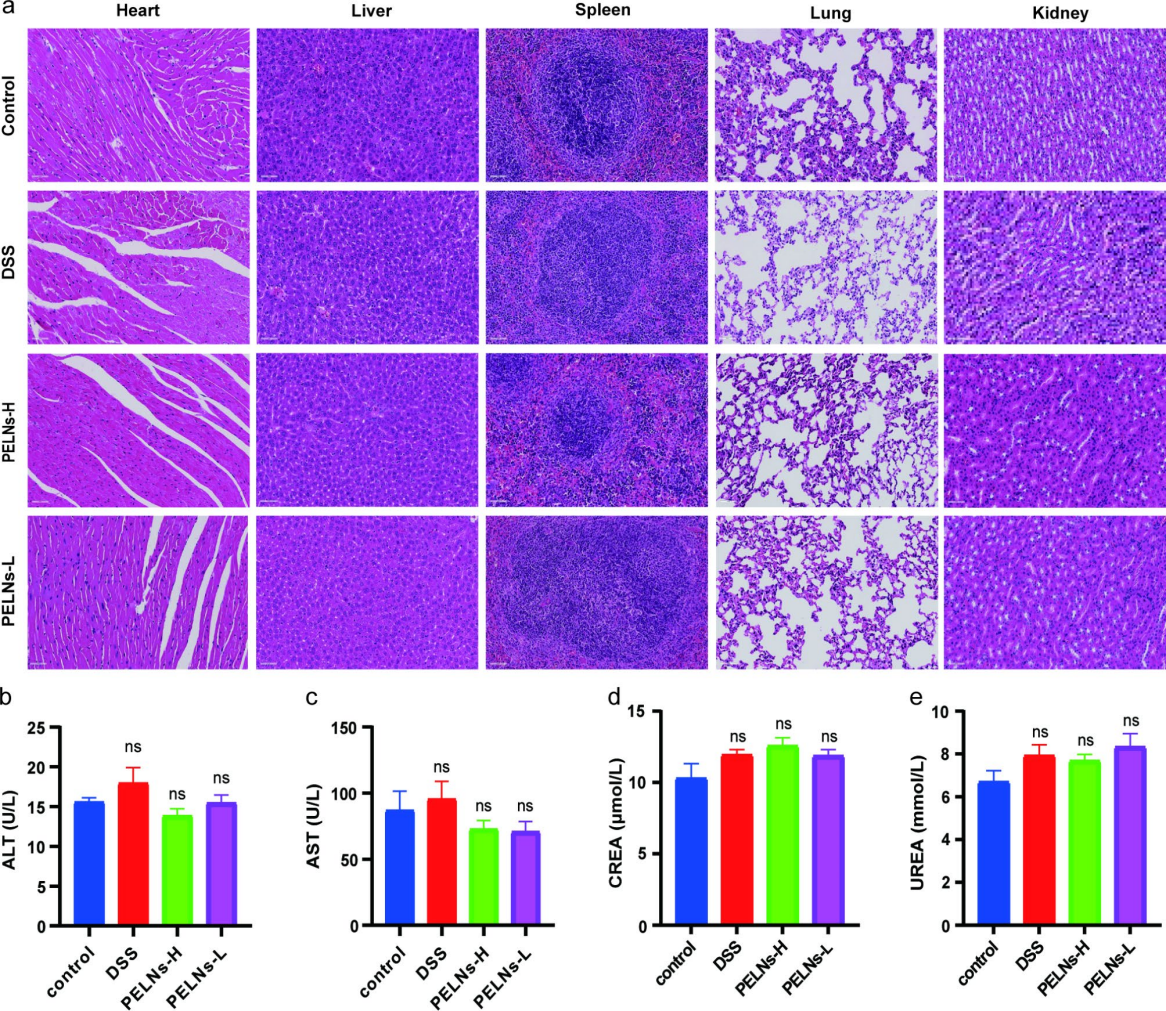
Biosafety of orally administered PELNs. **a**, H&E-stained of vital organs (heart, liver, spleen, lung, and kidney) in control, DSS, PELNs-L, and PELNs-H group; **b, c**, Liver function; **d, e**, Kidney function. ns p > 0.5

To assess the safety of orally administered PELNs, blood serum samples and vital organs (heart, liver, spleen, lung, and kidney) were collected from mice that received daily doses of 50 mg/g and 100 mg/g PELNs for 5 days. Histological analysis of the vital organs using H&E staining revealed no evident abnormalities or signs of organ damage in the PELNs-administered group (Fig. 8a). Furthermore, blood cell count, cardiac enzymes, glutamic pyruvic transaminase (ALT), aspartate aminotransferase (AST), serum creatinine (CREA), and serum urea (UREA) did not show significant changes between the normal control group, DSS group, and PELNs-administered group (Fig. 8b, c, d and e).

## Discussion

In recent years, there has been a growing interest in the use of exosome or exosome-like particle for the treatment of inflammatory diseases due to their unique physicochemical properties and targeted delivery capabilities [11, 20, 22, 40–45]. In this study, we isolated and identified PELNs derived from edible POL, which exhibited nanosized particles and negative zeta potential. Analysis of their components revealed the presence of various bioactive substances, including nucleic acids, lipids, and proteins. Furthermore, PELNs demonstrated the ability to suppress the expression of pro-inflammatory cytokines (IL-6, IL-12, IL-1β, and TNF-α) and MPO, and increase the levels of the anti-inflammatory cytokine IL-10, thereby alleviating DSS-induced colitis in mice. Additionally, PELNs exhibited excellent stability in simulated GIT conditions and showed specific targeting of the inflammatory site in mice with colitis. Mechanistically, PELNs treatment resulted in an increase in the abundance of *Lactobacillus reuteri* and indole derivatives. These indole derivatives activated the AhR in conventional CD4^+^ T cells, leading to the downregulation of Zbtb7b expression and the differentiation of conventional CD4^+^ T cells into DP CD4^+^CD8^+^ T cells. Based on these findings, our study suggested that oral administration of PELNs held promise as a potential therapeutic approach for colitis.

It is widely recognized that dysregulation in the composition and diversity of gut microbiota is implicated in the development of UC. UC is characterized by reduced α-diversity, depletion of *Firmicutes* and *Clostridium* cluster, and enrichment of *Actinobacteria* and *Proteobacteria*[46, 47]. A systematic review has shown a significant decrease in the abundance of *Eubacterium rectale* and *Akkermansia*, while an increase in levels of *E. coli* has been observed in UC patients [48]. Normally, gut microbiota metabolites, such as butyrate and short-chain fatty acids (SCFAs), play a crucial role in intestinal cell proliferation and have anti-inflammatory effect. Dysbiosis, on the other hand, leads to a decrease in the production of these metabolites, contributing to colitis [49, 50]. Fecal microbiota transplantation (FMT) has emerged as a promising alternative treatment for UC patients by increasing the abundance of SCFA-producing bacteria in the intestine, thus indirectly increasing the content of SCFAs, which effectively improves intestinal epithelial repair [51, 52]. Recent studies have indicated that PDENs can modulate the composition and function of gut microbiota, offering a novel therapeutic approach for UC patients. For instance, Zu et al. have demonstrated that edible “green” tea-derived nanoparticles can restore microbial homeostasis and alleviate colonic inflammation in a mouse model of colitis [22]. Similarly, edible ginger-derived exosome-like nanoparticles have been shown to be taken up by gut microbiota, maintaining microbiota balance and attenuating colitis in experimental mouse models [23]. In our present study, PELNs significantly increased the α-diversity and altered the structure of the intestinal microflora. The relative abundance of *Firmicutes* was notably elevated in mice treated with PELNs, while *Proteobacteria* showed a decrease. SCFA-producing bacteria, including *Lachnospiraceae_NK4A136_group*, *Lactobacillus*, *Ruminococcaceae_UCG-014*, *Ruminiclostridium_6*, *Lachnospiraceae_UCG-006*, *Ruminococcus_1*, and *Butyricicoccus*, were upregulated, while potentially pathogenic bacteria, including *Escherichia-Shigella*, *Enterococcus*, and *Parabacteroides*, were down-regulated. Overall, PELNs treatment restored the diversity of gut microbiota, decreased the abundance of harmful microbiota, and increased the abundance of probiotic bacteria.

*Escherichia-Shigella*, a harmful bacterium, has been associated with the etiology of IBD. Studies have shown that the abundance of *Escherichia-Shigella* is significantly elevated in patients with IBD compared to healthy controls [53]. However, reducing the abundance of *Escherichia-Shigella* has been found to protect the intestinal barrier and alleviate colitis [54]. In our present study, we observed a significant increase in the abundance of *Escherichia-Shigella* in the DSS group compared to the healthy control group, while the PELNs treatment dramatically decreased its abundance.

*Lactobacillus reuteri*, a probiotic bacterium, is naturally present in the human gut and has a wide range of beneficial effects on human health. Firstly, *Lactobacillus reuteri* is capable of inhibiting the colonization of pathogenic bacteria in its vicinity and can even remodel the gut microbiota through the production of antimicrobial molecules [55]. Secondly, *Lactobacillus reuteri* exhibits strong immunomodulatory and anti-inflammatory properties by promoting the development and functionality of regulatory T cells [56]. Thirdly, *Lactobacillus reuteri* can enhance the integrity of the gut barrier and reduce microbial translocation from the intestinal lumen to the tissues [57]. Impressively, *Lactobacillus reuteri* has shown promising potential as a therapeutic alternative for patients with UC [58]. In our current study, we discovered that PELNs treatment could improve the abundance of *Lactobacillus reuteri* both in vivo and in vitro.

Intestinal DP CD4^+^CD8^+^ T cells play an important role in immune tolerance and immune responses against gut antigens [59]. These cells also possess functions that include repression of intestinal inflammation, production of IL-10, and prevention of bacterial invasion [60]. DP CD4^+^CD8^+^ T cells originate from conventional CD4^+^ T cells, and their differentiation is regulated by the transcription factor Zbtb7b [61]. It is known that overexpression of Zbtb7b promotes the differentiation of conventional CD4^+^ T cells into CD4^+^ T cells, while downregulation of Zbtb7b induces the differentiation of conventional CD4^+^ T cells into DP CD4^+^CD8^+^ T cells [62]. Interestingly, our previous research has revealed that Zbtb7b is significantly upregulated in colonic tissue samples from UC patients and the DSS-induced colitis model. However, the abundance of DP CD4^+^CD8^+^ T cells in colon tissues was markedly decreased in the DSS-induced colitis group compared to the healthy control group [39]. Barragan et al. have demonstrated that *Lactobacillus reuteri* produces indole derivatives, which activate the AhR and downregulate the expression of Zbtb7b in conventional CD4^+^ T cells, thus promoting the reprogramming of conventional CD4^+^ T cells into DP CD4^+^CD8^+^ T cells [61]. In our present study, oral administration of PELNs significantly increased the levels of indole derivatives, effectively decreased the expression of Zbtb7b, and prominently facilitated the differentiation of DP CD4^+^CD8^+^ T cells in colonic tissues of mice with DSS-induced colitis. Therefore, we proposed that the oral administration of PELNs enhanced the abundance of *Lactobacillus reuteri* and increased the levels of indole derivatives, leading to the activation of AhR in conventional CD4^+^ T cells, subsequent downregulation of Zbtb7b, and increased differentiation of DP CD4^+^CD8^+^ T cells, ultimately mitigating colitis in mice.

Conventional therapeutic agents for UC have limited in clinical widespread application due to their potential systemic complications [7]. Although targeted biologic therapies have shown benefits for UC patients, they also come with disadvantages, such as high costs, non-responsiveness, and adverse events [7]. However, edible PDENs offer several advantages, including non-toxicity, non-immunogenicity, and abundant availability, making them a promising treatment option for UC [63, 64]. In our current study, oral administration of PELNs demonstrated excellent safety in the experimental mice.

In summary, our findings presented a novel and natural PELN derived from POL, which exhibited remarkable biosafety. Furthermore, oral administration of PELNs effectively reduced the expression of pro-inflammatory cytokines and increased the levels of anti-inflammatory cytokines. Importantly, the PELNs-*Lactobacillus reuteri*-indole-derivatives axis played a crucial role in downregulating the expression of Zbtb7b in conventional CD4^+^ T cells, resulting in the reprogramming of conventional CD4^+^ T cells into DP CD4^+^CD8^+^ T cells and subsequently alleviating colitis in mice. This important mechanism suggested that PELNs had the potential to be an effective alternative in the treatment of UC.

### Electronic supplementary material

Below is the link to the electronic supplementary material.

**Figure S1**. Physicochemical characterization of PELNs. a, Isolation and purification of PELNs from POL; b, Morphology of PELNs; c; The size distribution of PELNs; d, The average zeta potentials of PELNs; e, The percent of total PELNs lipids; f, The changes of PELNs zeta potential in mimicked a stomach-like solution and a small-intestine-like solution; g, The changes of PELNs sizes in mimicked a stomach-like solution and a small-intestine-like solution; h, PLNTs were labeled using an Odyssey fluorescent dye IRDye® 800CW NHS Ester. IRDye® 800CW-labeled PELNs suspended in PBS (a) or incubated in stomach-like solution(b), small intestine-like solution(c) at 37 ℃ for 30 min; i, The IRDye 800CW labeled PELNs were collected by exosome spin columns (MW4000), IRDye® 800CW-labeled PELNs were not weakened after incubation in both the stomach-like and small intestine-like solutions. **Figure S2**. Pro-inflammatory cytokines and anti-inflammatory cytokine expression profiles in IL-10^−/−^ mice. a, b, Colon length after oral administration of PELNs; c, d, e, f, qRT-PCR detecting the levels of IL-6, IL-12, IL-1β, and TNF-α in colonic samples; g, h, i, j, k, ELISA testing the expression profiles of IL-6, IL-12, IL-1β, TNF-α and MPO in blood serum. * *P* < 0.05, **P < 0.01, ***P < 0.001, ****P < 0.0001. **Figure S3**. DSS alters gut microbiota structure across different levels in mice. a-d, Welch’s t test analysis of DSS mediated differential microbial changes at the phylum, family, genus, and species level. **Figure S4**. The levels of indole derivatives after PELNs treatment. PELNs treatment can not change the levels of some indole derivatives, such as imdole, indoleadehtde, and indoleacrylic acid, in fecal samples as compared to the DSS group, whereas PELNs treatment decrease the level of indoleacetic acid compared to the DSS group. **Table S1** The primer sequences.

## Data Availability

All data generated or analyzed during this study are included in this manuscript and its additional files.
