# Supplementary material for: Edible exosome-like nanoparticles from portulaca oleracea L mitigate DSS-induced colitis via facilitating double-positive CD4+CD8+T cells expansion
Source: J Nanobiotechnology. 2023 Aug 31;21:309. doi: 10.1186/s12951-023-02065-0 (PMC10469825; doi:10.1186/s12951-023-02065-0)
Supplement: Supplementary file 1 — Figure S1. Physicochemical characterization of PELNs. a, Isolation and purification of PELNs from POL; b, Morphology of PELNs; c; The size distribution of PELNs; d, The average zeta potentials of PELNs; e, The percent of total PELNs lipids; f, The changes of PELNs zeta potential in mimicked a stomach-like solution and a small-intestine-like solution; g, The changes of PELNs sizes in mimicked a stomach-like solution and a small-intestine-like solution; h, PLNTs were labeled using an Odyssey fluorescent dye IRDye® 800CW NHS Ester. IRDye® 800CW-labeled PELNs suspended in PBS (a) or incubated in stomach-like solution(b), small intestine-like solution(c) at 37 ℃ for 30 min; i, The IRDye 800CW labeled PELNs were collected by exosome spin columns (MW4000), IRDye® 800CW-labeled PELNs were not weakened after incubation in both the stomach-like and small intestine-like solutions. Figure S2. Pro-inflammatory cytokines and anti-inflammatory cytokine expression profiles in IL-10−/− mice. a, b, Colon length after oral administration of PELNs; c, d, e, f, qRT-PCR detecting the levels of IL-6, IL-12, IL-1β, and TNF-α in colonic samples; g, h, i, j, k, ELISA testing the expression profiles of IL-6, IL-12, IL-1β, TNF-α and MPO in blood serum. * P < 0.05, **P < 0.01, ***P < 0.001, ****P < 0.0001. Figure S3. DSS alters gut microbiota structure across different levels in mice. a-d, Welch’s t test analysis of DSS mediated differential microbial changes at the phylum, family, genus, and species level. Figure S4. The levels of indole derivatives after PELNs treatment. PELNs treatment can not change the levels of some indole derivatives, such as imdole, indoleadehtde, and indoleacrylic acid, in fecal samples as compared to the DSS group, whereas PELNs treatment decrease the level of indoleacetic acid compared to the DSS group. Table S1 The primer sequences. [file 12951_2023_2065_MOESM1_ESM.docx]

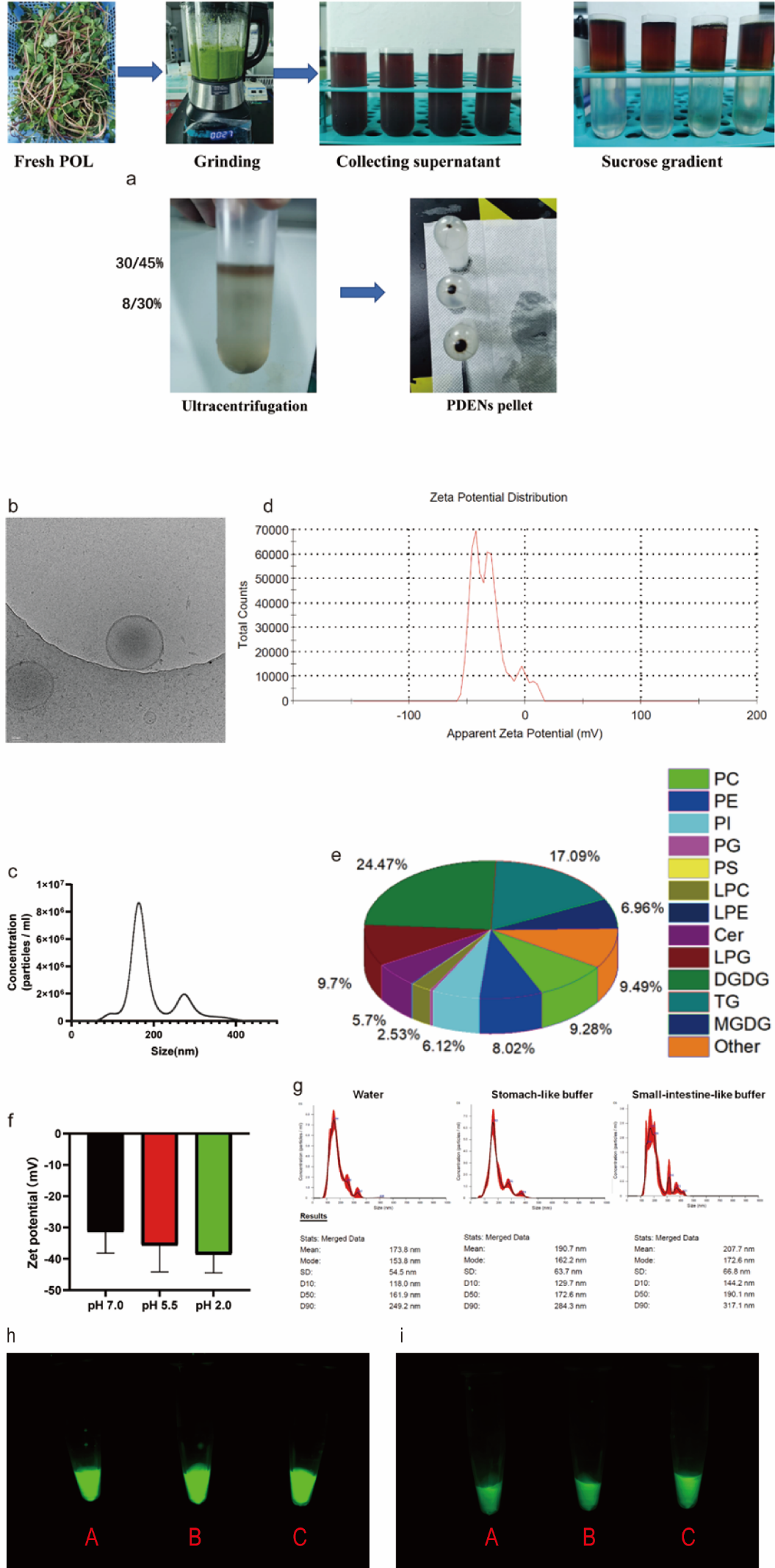


Figure S1: **Physicochemical characterization of PELNs.** a, Isolation and purification of PELNs from POL; b, Morphology of PELNs; c; The size distribution of PELNs; d, The average zeta potentials of PELNs; e, The percent of total PELNs lipids; f, The changes of PELNs zeta potential in mimicked a stomach-like solution and a small-intestine-like solution; g, The changes of PELNs sizes in mimicked a stomach-like solution and a small-intestine-like solution; h, PLNTs were labeled using an Odyssey fluorescent dye IRDye® 800CW NHS Ester. IRDye® 800CW-labeled PELNs suspended in PBS (a) or incubated in stomach-like solution(b), small intestine-like solution(c) at 37 ℃ for 30 min; i, The IRDye 800CW labeled PELNs were collected by exosome spin columns (MW4000), IRDye® 800CW-labeled PELNs were not weakened after incubation in both the stomach-like and small intestine-like solutions.


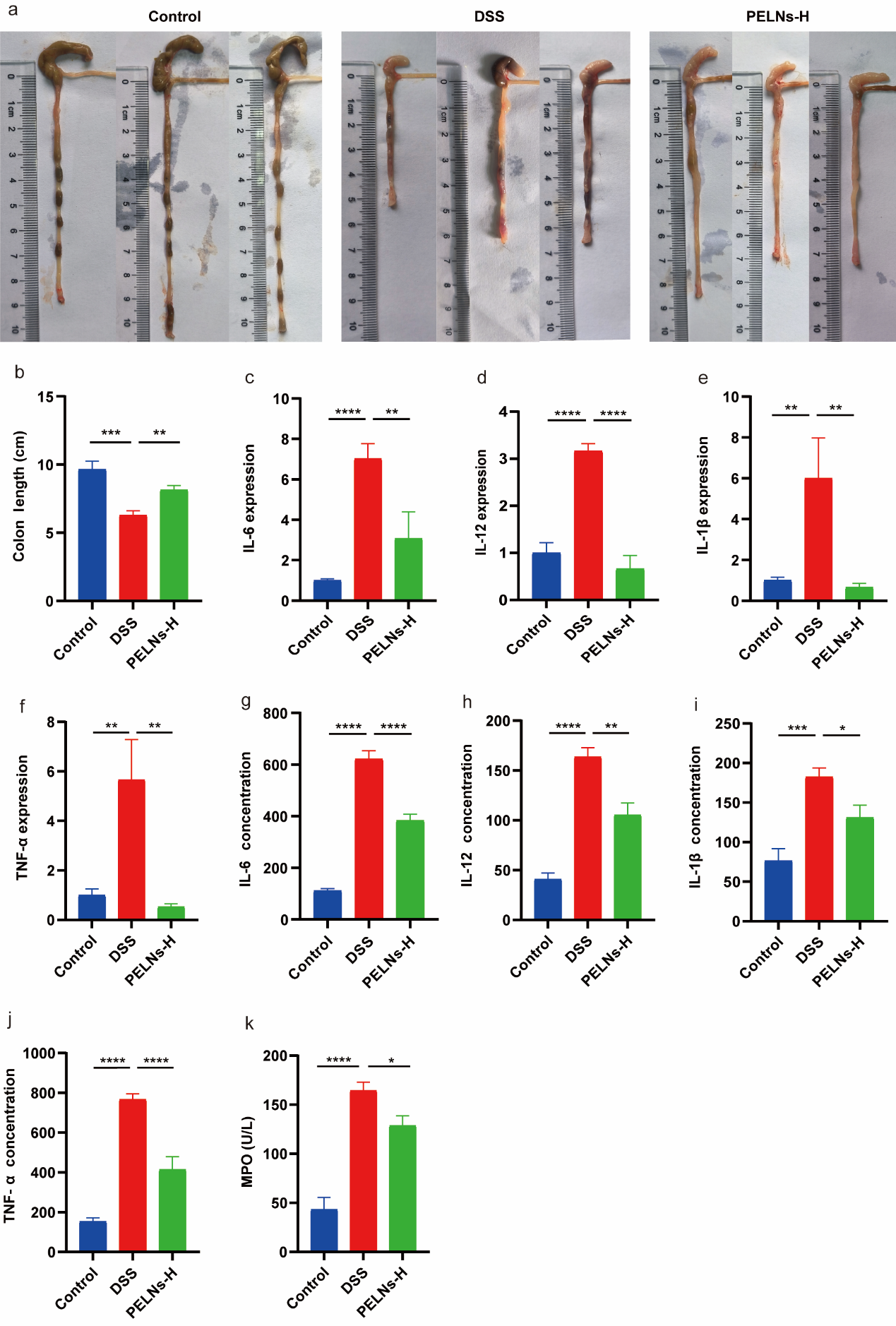


Figure S2: **Pro-inflammatory cytokines and anti-inflammatory cytokine expression profiles in** **IL-10^-/-^ mice.** a, b, Colon length after oral administration of PELNs; c, d, e, f, qRT-PCR detecting the levels of IL-6, IL-12, IL-1β, and TNF-α in colonic samples; g, h, i, j, k, ELISA testing the expression profiles of IL-6, IL-12, IL-1β, TNF-α and MPO in blood serum. * *P*<0.05, ***P*<0.01, ****P*<0.001, *****P*<0.0001.


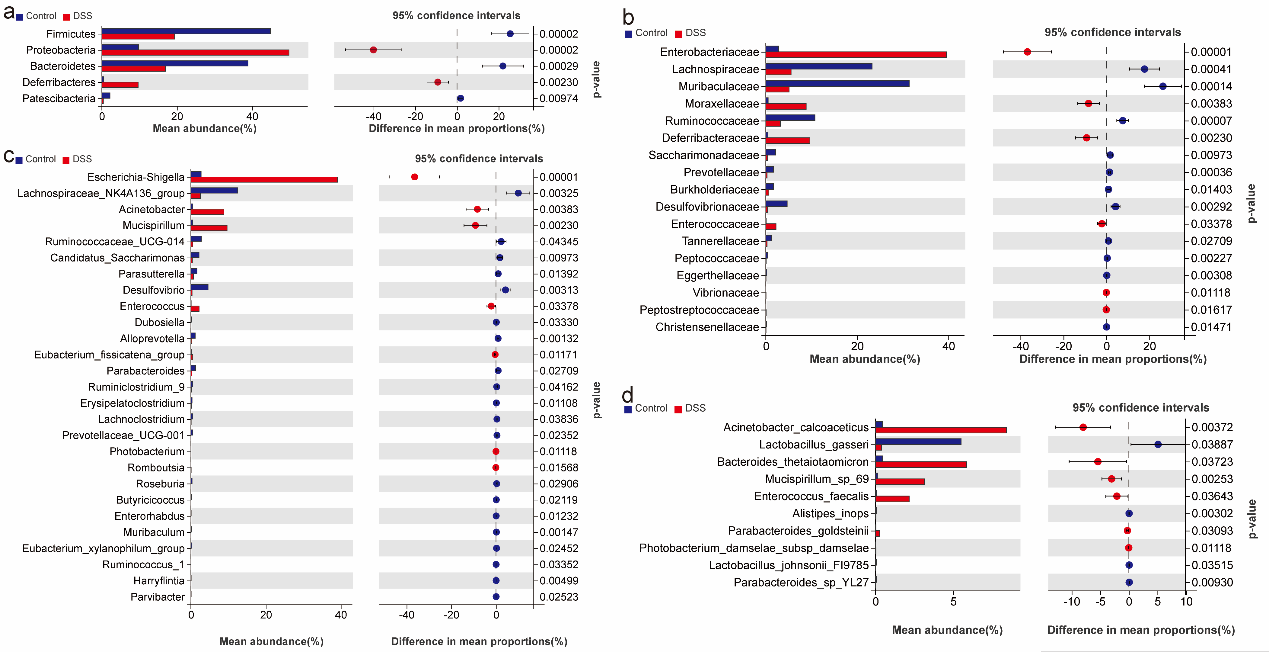


**Figure S3: DSS alters gut microbiota structure across different levels in mice.** a-d, Welch's t test analysis of DSS mediated differential microbial changes at the phylum, family, genus, and species level.


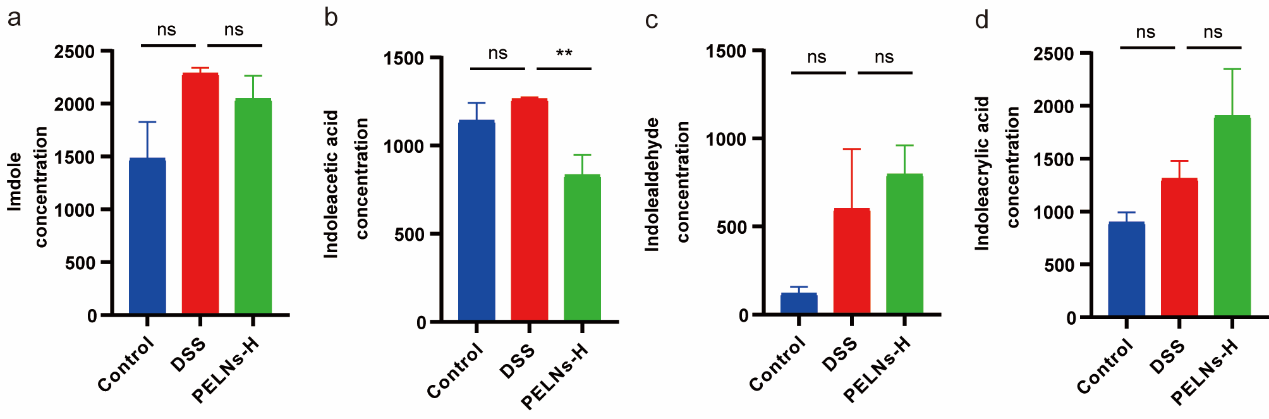


Figure S4: **The levels of indole derivatives after PELNs treatment**. PELNs treatment can not change the levels of some indole derivatives, such as imdole, indoleadehtde, and indoleacrylic acid, in fecal samples as compared to the DSS group, whereas PELNs treatment decrease the level of indoleacetic acid compared to the DSS group.

Table S1 The primer sequences.

| Gene | Forward | Reverse |
| --- | --- | --- |
| GAPDH (mouse) | 5'- CATCACTGCCACCCAGAAGACTG-3' | 5'- ATGCCAGTGAGCTTCCCGTTCAG-3' |
| IL-6  (mouse) | 5'- TACCACTTCACAAGTCGGAGGC-3' | 5'- CTGCAAGTGCATCATCGTTGTTC-3' |
| IL-10  (mouse) | 5'-CGGGAAGACAATAACTGCACCC-3' | 5'-CGGTTAGCAGTATGTTGTCCAGC-3' |
| IL-12  (mouse) | 5'-TTGAACTGGCGTTGGAAGCACG-3' | 5'-CCACCTGTGAGTTCTTCAAAGGC-3' |
| IL-1β  (mouse) | 5'-TGGACCTTCCAGGATGAGGACA-3' | 5'-GTTCATCTCGGAGCCTGTAGTG-3' |
| TNF-α  (mouse) | 5'-GGTGCCTATGTCTCAGCCTCTT-3' | 5'-GTTCATCTCGGAGCCTGTAGTG-3' |
